# Overcoming Linsitinib intrinsic resistance through inhibition of nuclear factor‐*κ*B signaling in esophageal squamous cell carcinoma

**DOI:** 10.1002/cam4.1068

**Published:** 2017-04-24

**Authors:** Junzhou Wu, Kaiyan Chen, Fanrong Zhang, Jiaoyue Jin, Nan Zhang, Dan Li, Lisha Ying, Wei Chen, Herbert Yu, Weimin Mao, Dan Su

**Affiliations:** ^1^ Cancer Research InstituteZhejiang Cancer Hospital & Key Laboratory Diagnosis and Treatment Technology on Thoracic Oncology of Zhejiang ProvinceHangzhouChina; ^2^ Department of OncologyThe Second Clinical Medical College of Zhejiang Chinese Medical UniversityHangzhouChina; ^3^ Cancer Epidemiology ProgramUniversity of Hawaii Cancer CenterHawaiiUSA

**Keywords:** Esophageal cancer, IGF‐1R, intrinsic resistance, NF‐*κ*B p65, targeted therapy

## Abstract

The aim of this study is to evaluate the efficacy of insulin‐like growth factor 1 receptor (IGF‐1R) inhibitor Linsitinib, in esophageal squamous cell carcinoma (ESCC), and to characterize special biomarker to screen Linsitinib‐sensitive patients as well as explore the molecular‐resistant mechanism to Linsitinib in ESCC. Our study evaluated the sensitivity of insulin‐like growth factor 1 receptor (IGF‐1R) inhibitor, Linsitinib in ESCC cells with MTT assay. After Linsitinib treatment, the expressions of downstream signaling molecules and apoptosis pathways were measured by western blot. And the antitumor effect of Linsitinib and JSH‐23, an inhibitor of nuclear factor‐*κ*B transcriptional activity, was analyzed both as single agent and in combination in ESCC. Apoptosis, cell viability, and clonogenic survival analysis were also investigated. The sensitivity of Linsitinib was relatively variable in patient‐derived primary ESCC cells as well as in human commercial cell lines. And the downstream AKT/mTOR and ERK signaling pathways were inhibited by Linsitinib, while phosphorylation level of NF‐*κ*B p65 was obviously activated to reduce apoptosis effect in Linsitinib‐resistant cell lines. Most importantly, blockage of NF‐*κ*B activity by JSH‐23 could sensitize resistant cells to Linsitinib treatment. Results from this study demonstrated that the intrinsic resistance to Linsitinib was predominantly mediated by NF‐*κ*B activation in ESCC. Moreover, combination of Linsitinib and JSH‐23 as therapy provides a novel strategy to overcome resistance to Linsitinib in ESCC.

## Introduction

Esophageal cancer is one of the most aggressive and highly lethal gastrointestinal tract malignancies 1, 2. Esophageal squamous cell carcinoma (ESCC) accounts for more than 90% of esophageal cancer cases in China 3. Despite the rapid advance in tumor‐targeted therapeutics, no new regimens are effective in ESCC 4, and the 5‐year overall survival rate for ESCC remains dismal 5, 6.

Insulin‐like growth factor‐1 receptor (IGF‐1R) signaling pathway has been implicated in the carcinogenesis and progression of multiple cancer sites, including ESCC 7, 8. Studies of ESCC demonstrated that upon binding to its ligands IGF‐1 or IGF‐2, IGF‐1R is autophosphorylated and the phosphorylation activates the downstream pathways of PI3K/AKT/mTOR and Ras/Raf/MEK/MAPK 9, 10, which promote tumor cell proliferation, invasion, metastasis, and evasion of apoptosis 10, 11. Moreover, elevated levels of IGF‐1R expression are common in 60–80% ESCC 12, 13, and patients with higher expression of IGF‐1R are more likely to have shorter overall survival 7. Thus, inhibition of the IGF‐1R pathway may offer a promising strategy for ESCC treatment.

Recently, around 30 compounds targeting IGF‐1R have been tested in phase II/III clinical trials for the treatment of several types of cancer including ESCC. 8, 14, 15, 16. Among them, Linsitinib (also named OSI‐906) is a selective and orally bioavailable IGF‐1R/insulin receptor (IR) inhibitor 17, which has been shown to block ligand‐induced activation of pAkt, pERK1/2, and p‐p70S6K 15. However, clinical trials involving Linsitinib showed varied response rates 18, 19, 20. Two phase I trials showed an overall objective response rate of about 30% in advanced solid tumors 18, 19, with some patients obtaining durable benefit from the IGF‐1R blockage 18, 19. However, a phase III clinical trial of adrenocortical carcinoma indicated that Linsitinib had no effect in comparison to the placebo group 21. The different responses may be partly due to the innate drug resistance or activation of compensatory pathways allowing for continued growth 15, 22. To deal with these challenges, we need to elucidate the mechanisms that underlie Linsitinib resistance in ESCC and identify biomarkers that can screen Linsitinib‐sensitive patients with ESCC.

In this study, we investigated the mechanisms underpinning the sensitivity and resistance of Linsitinib in ESCC, and found an intrinsic Linsitinib resistance mediated through the nuclear factor‐*κ*B (NF‐*κ*B) pathway. Our experiments suggest that Linsitinib administration in combination with NF‐*κ*B inhibitor JSH‐23 may have synergy in ESCC treatment.

## Methods

### Ethics approval

This study was approved by the institutional review board of Zhejiang Cancer Hospital. All patients signed an informed consent before surgery.

### Cell lines and cell culture

Human commercially available ESCC cell lines were bought from Chinese Academy of Sciences, Shanghai Institutes for Biological Sciences (Shanghai, China). The patient‐derived primary cancer cells were isolated and cultured from solid tumors of ESCC patients. Reduction esophagectomy tissue samples were mechanically dissociated and then incubated with collagenase (Roche Life Science, Indianapolis, IN) and hyaluronidase (Sigma‐Aldrich, St. Louis, MO) at 37°C for 2 h. Primary cancer cells thus obtained were tested for drug response between passage 3 to 5 generations. All tissue specimens used in our study were obtained from the tissue bank of Zhejiang Cancer Hospital. All patients signed an informed consent before surgery. This study was approved by the institutional review board of Zhejiang Cancer Hospital.

Primary ESCC cells were cultured in DMEM/F12 (Life technologies, Gaithersburg, MD). DMEM/F12 medium was supplemented with 10% fetal bovine serum (FBS, Gibco, Life technologies), 1% penicillin‐streptomycin (Gibco, Life technologies), and 1% MEM nonessential amino acids (Gibco, Life technologies). All other cells were cultured in RPMI 1640 (Life technologies), supplemented with 10% FBS. All the cells were cultured under the standard conditions (5% CO_2_ at 37°C).

### Antibodies and reagents

Linsitinib and JSH‐23 were purchased from Selleckchem Co. (Houston, TX). Stock solutions with a concentration of 10 mM were prepared and stored at −20°C.

Antibodies against phospho‐IGF1R (CST‐3918), total‐IGF1R (CST‐9750), phospho‐p65 (CST‐3033), total‐p65 (CST‐8242), phospho‐Akt308 (CST‐2965), phospho‐Akt473 (CST‐9271), total‐Akt (CST‐1085), phospho‐mTOR (CST‐5536), total‐mTOR (CST‐2983), phospho‐ERK1/2 (CST‐9102), total‐ERK1/2 (CST‐4376), and PARP (CST‐1078) were purchased from Cell Signaling Technology (Danvers, MA, USA). Cleaved Caspase‐3 (25546‐1‐AP) were purchased from Protein Technology (Tucson, AZ) and Tubulin (ARH4207) were purchased from AR (San Diego, CA). HRP‐conjugated goat anti‐mouse and goat anti‐rabbit antibodies were from Santa Cruz Biotechnology (Dallas, TX). MTT [3‐(4,5‐dimethylthiazol‐2‐yl)‐2,5‐diphenyl‐2H‐tetrazolium bromide] was obtained from Sigma‐Aldrich.

### Cell viability analysis

A colorimetric MTT assay was performed to quantify the effect of drugs on cell viability. Cells were seeded in 96‐well plates at a density of 3000 cells/well, and were grown for over 24 h before being incubated with the respective compound for 72 h. Controls were treated with DMSO only. Four hours prior to the end of the culture period, 50 *μ*L MTT solution (5 mg/mL in PBS) was added to each well. Each reaction was stopped by adding 150 *μ*L DMSO. The absorbance was measured at a wavelength of 570 nm.

### Cell lysis and western blot

Cells were lysed to extract proteins with a lysis buffer using the standard method. The extracted protein samples were analyzed using 8% or 12% SDS‐polyacrylamide gel electrophoresis (SDS‐PAGE), and the separated proteins were transferred onto polyvinylidene difluoride membranes (Millipore, Bedford, MA). Each membrane was blocked with 5% nonfat dry milk or bovine serum albumin in TBS‐Tween‐20 (TBS‐T) for 1 h, followed by incubation with primary antibody at 4°C overnight. The membrane was then incubated with HRP‐conjugated secondary antibody before it was detected with enhanced chemiluminescence (ECL, Millipore).

### RNA extraction and quantitative real‐time PCR

Total RNA was extracted from cells with the TRIzol reagent and cDNA was synthesized using a Prime‐Script RT Reagent kit (Takara Bio, Inc. Otsu, Shiga, Japan). Targeted cDNA were amplified using a SYBR Premix Ex Taq kit (Takara Bio) on ABI 7500 Real‐time polymerase chain reaction (PCR) System (Life Technology, Foster City, CA). PCR condition was one cycle at 50°C for 2 min and then 40 cycles of 5 sec at 95°C and 35 sec at 60°C. The sequences of PCR primers used were as follows: IL‐6, 5′‐TTCCATCCAGTTGCCTTCTT‐3′ (forward), and 5′‐CAGAATTGCCATTGCACAAC‐3′ (reverse); IL‐8, 5′‐ATGACTTCCAAGCTGGCCGTGGCT‐3′ (forward), and 5′‐TCTCAGCCCTCTTCAAAAACTTCTC‐3′ (reverse); GAPDH, 5′‐GAAGGTGAAGGTCGGAGTC‐3′ (forward) and 5′‐GAAGATGGTGATGGGATTTC‐3′(reverse). The results were normalized against GAPDH expression. Levels of gene expression were calculated using the formula 2^−ΔΔCt^.

### Flow cytometric analysis of apoptosis cell

Cells (1 × 10^5^/mL) were seeded in 6‐well plates and incubated overnight. The cells were than exposed to Linsitinib (1.0 or 10.0 *μ*mol/L) and JSH‐23 (20 *μ*mol/L) alone or in combination for 48 h. After the treatment, cells were stained with the Annexin V‐FITC Apoptosis Detection kit (Sigma‐Aldrich) for 5 min at 4°C in dark. Apoptotic cells were measured with a FACS Calibur flow cytometer, and the results were analyzed by CellQuest software (BD Biosicences, San Diego, CA).

### Colony formation assay

Cells (1 × 10^3^ cells per plate) were seeded onto 6‐well plates and then treated with Linsitinib (1.0 or 10.0 *μ*mol/L) and JSH‐23 (20 *μ*mol/L) alone or in combination for at least 10 days. At the end of the incubation period, cells were fixed with methanol and stained with 0.05% crystal violet solution. Colonies containing >50 cells were counted.

### Statistical analysis

Statistical analyses were performed using SPSS (version 18.0, Chicago, IL). Data from the experiments were expressed as mean ± SD which was based on a minimum of three independent experiments. Differences between groups were compared using the two‐way ANOVA, followed by the Newman–Keuls test, and a *P* < 0.05 was considered significant.

## Results

### Effect of Linsitinib on ESCC cells

**Figure 1:**
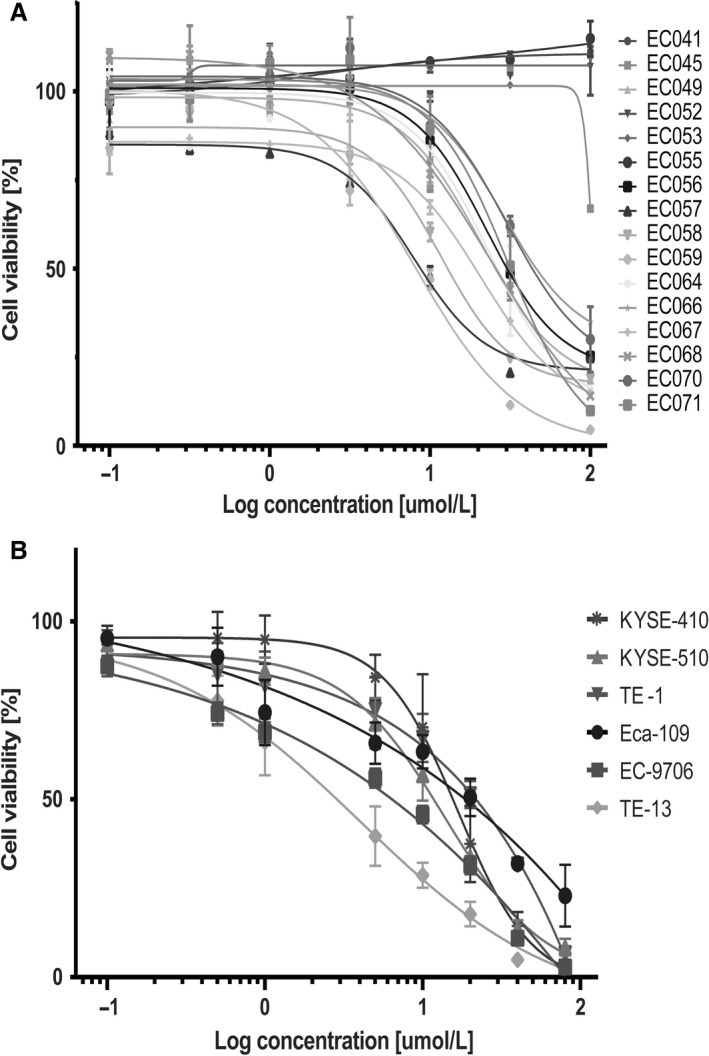
Sensitivity of Linsitinib in ESCC cells. (A) A panel of 16 patient‐derived primary ESCC cells was seeded in growth medium containing different concentrations of Linsitinib (0.1–100 *μ*mol/L) for 72 h. Then MTT assay was performed to measure the cell viability. (B) The sensitivity of Linsitinib with concentrations of 0.1–80 *μ*mol/L was also evaluated in another panel of six commercially available ESCC cell lines including Eca‐109, EC‐9706, KYSE‐510, KYSE‐410, TE‐1, and TE‐13. ESCC, esophageal squamous cell carcinoma.

To investigate the effect of Linsitinib on the viability of ESCC cells, a panel of 16 primary ESCC cells was exposed to different concentrations of Linsitinib (0.1–100 *μ*mol/L), and the cell viability was then measured with MTT assay (Fig. 1A). The test results showed that four of 16 primary cells were almost completely resistant to Linsitinib, while four displayed high sensitivity. In addition, we evaluated the sensitivity of Linsitinib with concentrations of 0.1–80 *μ*mol/L in a panel of six commercially available ESCC cell lines (Eca‐109, EC‐9706, KYSE‐510, KYSE‐410, TE‐1, and TE‐13). As shown in Figure 1B, TE‐13 was the most sensitive cell line and the remaining five were much resistant. The sensitive (TE‐13) and resistant cell lines (TE‐1 and KYSE‐510) were further examined for drug resistance.

### Inhibition of ERK and PI3K signaling by Linsitinib

**Figure 2:**
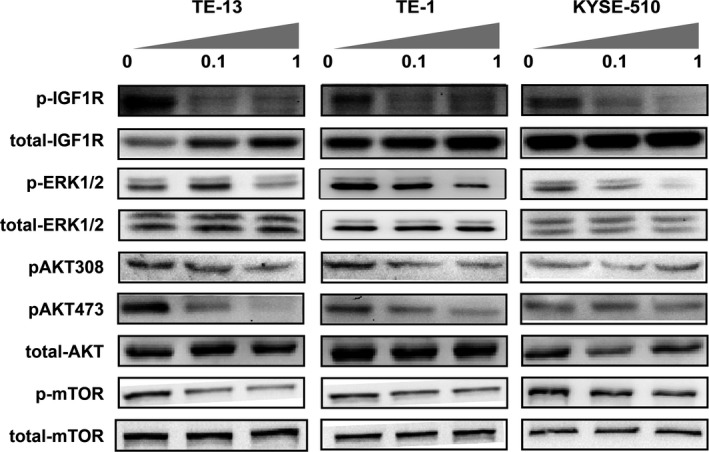
Linsitinib inhibits the activation of ERK and PI3K pathways. After treatment of Linsitinib at 0.1 or 1.0 *μ*mol/L for 72 h, the phosphorylation of insulin‐like growth factor‐1 receptor (IGF‐1R), ERK1/2, and AKT/mTOR pathways were analyzed in TE‐13, TE‐1, and KYSE‐510 cell lines by western blot.

Since incomplete blockade of the IGF‐1R pathway could confer Linsitinib resistance, we evaluated the downstream effectors of the MEK and PI3K pathways (ERK1/2, AKT and mTOR) with western blot. As shown in Figure 2, IGF‐1R phosphorylation was inhibited after treatment of Linsitinib at 0.1 or 1.0 *μ*mol/L for 72 h. Moreover, compared to the controls, phosphorylation of ERK1/2 was down‐regulated by Linsitinib in both sensitive (TE‐13) and resistant cell lines (TE‐1, KYSE‐510), and levels of phosphorylated AKT and mTOR were low in all three cell lines. These results suggested that Linsitinib could effectively inactivate two major downstream pathways of IGF‐1R.

### Reduced apoptosis in Linsitinib‐resistant ESCC cells

**Figure 3:**
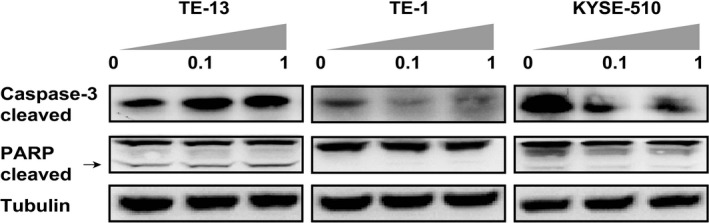
Reduced apoptosis in Linsitinib‐resistant esophageal squamous cell carcinoma cells. After treatment of Linsitinib at 0.1 or 1.0 *μ*mol/L for 72 h, cleaved PARP and activated Caspase‐3 were examined in TE‐13, TE‐1, and KYSE‐510 cells by western blot. Tubulin was used as a loading control.

To examine if Linsitinib resistance affects cell apoptosis, we analyzed the expression of cleaved PARP and activated Caspase‐3 protein with western blot. As indicated in Figure 3, after treatment of Linsitinib at 0.1 or 1.0 *μ*mol/L for 72 h, the expression of cleaved PARP and activated Caspase‐3 were increased in a sensitive cell line (TE‐13), but decreased in resistant cell lines (TE‐1 and KYSE‐510). These results suggested that Linsitinib‐resistant cells had a reduced capacity of apoptosis.

### Activation of NF‐*κ*B pathway in Linsitinib‐resistant ESCC cells

**Figure 4:**
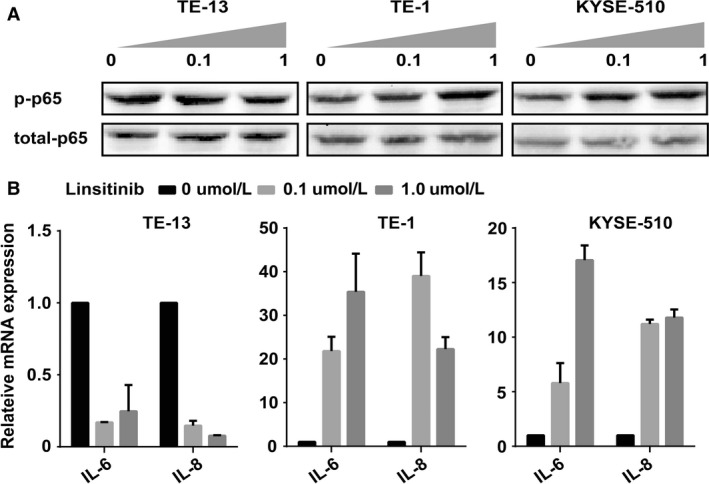
Activation of nuclear factor‐*κ*B (NF‐*κ*B) pathway in Linsitinib‐resistant esophageal squamous cell carcinoma cells. (A) With the treatment of the different concentrations of Linsitinib (0, 0.1, and 1.0 *μ*mol/L) for 72 h, the phosphorylation of NF‐*κ*B p65 was evaluated in TE‐13, TE‐1, and KYSE‐510 cells by western blot. (B) IL‐6 and IL‐8 mRNA levels were measured by real‐time polymerase chain reaction; untreated cells were included as controls. Every experiment was performed at least three times in triplicate determinations. Each result was corrected by value from a control study.

NF‐*κ*B pathway may play a critical role in drug resistance of ESCC cells. We evaluated whether NF‐*κ*B pathway was activated after Linsitinib treatment. Figure 4A shows a dose‐dependent elevation of p‐p65 levels in Linsitinib‐resistant cell lines (TE‐1 and KYSE‐510), with little or no change in total p65 levels. Expression of p‐p65 was inhibited in TE‐13, the Linsitinib‐sensitive cell line. Additionally, as the transcriptional targets of p65, IL‐6 and IL8 mRNA levels were also altered following the trends of p‐p65, up‐regulation in resistant cell lines, and down‐regulation in the sensitive cell line (Fig. 4B). Taken together, our experiment suggested that NF‐*κ*B pathway was activated in resistant ESCC cells after Linsitinib treatment.

### Enhanced apoptosis following combined treatment of Linsitinib and NF‐*κ*B inhibitor in Linsitinib‐resistant cells

**Figure 5:**
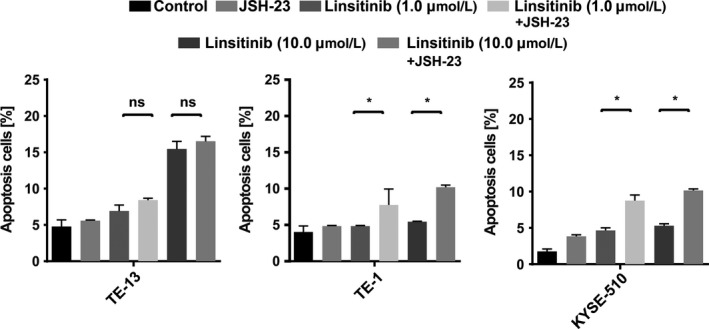
Apoptosis was enhanced following the combination of Linsitinib and nuclear factor‐*κ*B (NF‐*κ*B) inhibitor in Linsitinib‐resistant cells. TE‐13, TE‐1, and KYSE‐510 cell lines were seeded in growth medium with Linsitinib (1.0 or 10.0 *μ*mol/L) and JSH‐23 (20 *μ*mol/L) alone or in combination for 48 h. Then cells were harvested and the Annexin V‐FITC apoptosis assay was performed to measure the percentage of apoptotic cells. And results from three independent experiments are summarized in Figure 5. NS indicates *P *>* *0.05. *P <* *0.01

To test if NF‐*κ*B inhibitor could be used in combination with IGF‐1R blocker in treating ESCC, we performed flow cytometry analysis of cell apoptosis in Linsitinib ‐sensitive and ‐resistant cells (Fig. 5). Interestingly, after exposing cells to Linsitinib (1.0 or 10.0 *μ*mol/L) and JSH‐23 (20 *μ*mol/L) alone or in combination for 48 h, cells treated with the combined regimens showed statistically significant induction of programmed cell death when compared to single‐regimen treatment in Linsitinib‐resistant cells (*P *<* *0.01, Fig. 5). However, this effect was not observed in Linsitinib‐sensitive cell TE‐13 (*P *>* *0.05, Fig. 5). This difference suggests that reduction in apoptosis may be an important mechanism in tumors resistant to Linsitinib.

### Combined treatment of Linsitinib and NF‐*κ*B inhibitor affected cell viability and colony formation in Linsitinib‐resistant cells

**Figure 6:**
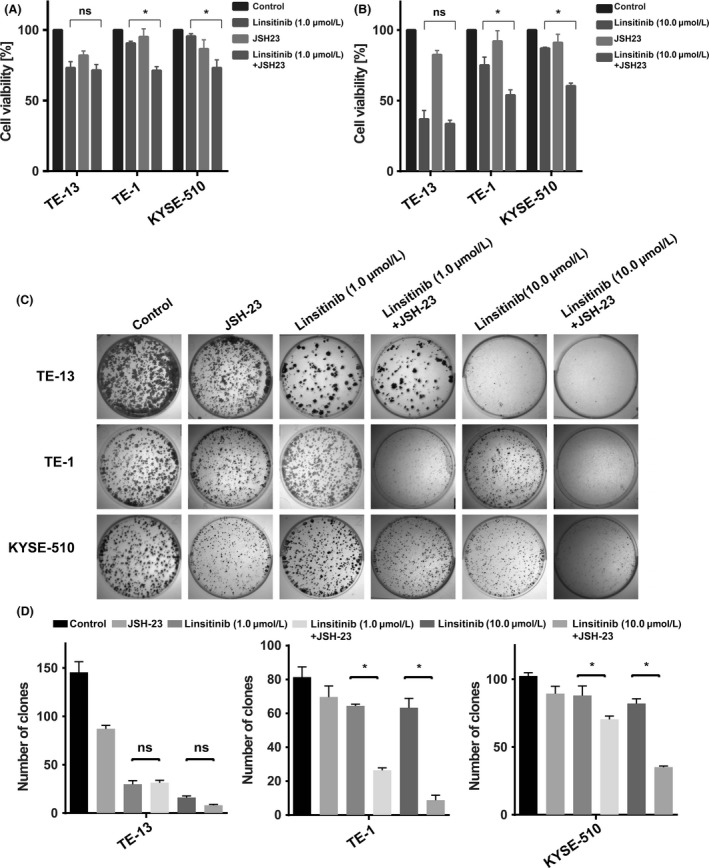
Combined treatment of Linsitinib and nuclear factor‐*κ*B (NF‐*κ*B) inhibitor affected cell viability and colony formation ability in Linsitinib‐resistant cells. TE‐13, TE‐1, and KYSE‐510 cells were exposed to Linsitinib 1.0 *μ*mol/L for (A), 10.0 *μ*mol/L for (B), and JSH‐23 (20 *μ*mol/L) alone or in combination. Then MTT assay was performed to measure the cell viability, and data from each cell line represent mean growth inhibition compared to DMSO control cells for three independent experiments. Furthermore, colony formation analysis was also investigated. Representative images are shown in (C) and results from three independent experiments are summarized in (D). NS indicates *P *>* *0.05. *P <* *0.01

We next measured cell viability and colony formation ability after exposing the cells to Linsitinib (1.0 or 10.0 *μ*mol/L) and JSH‐23 (20 *μ*mol/L) alone or in combination (Fig. 6A). As depicted in Fig. 6A, compared to Linsitinib monotherapy, the combination of Linsitinib and JSH‐23 had a statistically significant effect on growth inhibition of Linsitinib‐resistant cells (TE‐1 and KYSE‐510, *P *<* *0.01). However, no inhibitory effect was observed in Linsitinib‐sensitive cell (TE‐13, *P *>* *0.05). Similarly, the addition of JSH‐23 could reverse ESCC cells from Linsitinib resistant to Linsitinib sensitive with regard to their colony formation.

## Discussion

Recent clinical trials of IGF‐1R inhibitors have demonstrated variable antitumor effects 20, 23, which may reflect either the lack of patient selection strategies and/or little understanding of drug‐resistant mechanisms. Consistent with the findings of a previous study of Linsitinib in colorectal cancer 24, our study also showed that the sensitivity of Linsitinib was variable not only in primary cells but also in commercial cell lines. This observation indicates to the presence of intrinsic resistance to Linsitinib in ESCC. Possible mechanisms explaining the intrinsic resistance include limited effect on downstream signaling of IGF‐1R, existence of subclones resistant to the drug, and alternative compensatory pathway 25, 26. Previous studies showed that cell lines with active downstream molecules MAPK/MEK 27, 28 or AKT/mTOR/p70S6K 24, 29 had intrinsic resistance to IGF‐1R inhibitor. Moreover, using Linsitinib in combination with a MEK inhibitor to treat colorectal cancer cells with active MAPK demonstrated synergistic antitumor effects on the Linsitinib‐resistant cell lines 27. In our study, we found that the AKT/mTOR and ERK1/2 pathways were inhibited by Linsitinib in both sensitive and resistant cell lines. On the basis of this observation, we hypothesized that failure to Linsitinib treatment was not due to the activities of IGF‐1R downstream molecules, but rather resulted from a compensatory mechanism that counteracted the effect of a single regimen which targeted only upstream molecule.

As previously proposed, IGF‐1R inhibitors could induce apoptosis, inhibit tumor growth, as well as sensitize cells to chemotherapy in esophageal carcinoma cells 29, 30. Programmed cell death can be suppressed by the nucleus localization of nuclear factor‐*κ*B (NF‐*κ*B) 31, which induces the expression of antiapoptotic factors such as the IAPs, the TRAFs, and Bfl‐1 32. NF‐*κ*B plays a critical role in chemotherapy resistance due to its ability to reduce apoptosis 33, 34, 35, 36, 37. Apoptotic pathways are related with the sensitivity of target drugs 38, 39, 40, and Linsitinib resistance in ESCC may be related to apoptosis. So we investigated the expression of cleaved PARP, activated Caspase‐3, and phosphorylated NF‐*κ*B p65, as well as its transcriptional targets IL‐6 and IL8. Interestingly, our results demonstrated that the apoptotic effect was decreased, while NF‐*κ*B p‐p65 was significantly increased in Linsitinib‐resistant cells. Meanwhile, the opposite trend was observed in Linsitinib‐sensitive cells.

To further confirm these results, we investigated the combined effects of Linsitinib and JSH‐23, a molecule that inhibits the transcriptional activity of NF‐*κ*B, on ESCC cell growth. JSH‐23 has been found to reduce the resistance to TRAIL‐induced apoptosis in acute myeloid leukemia 41. In addition, JSH‐23 has been proven to reverse the radioresistance in breast cancer 42. We investigated the apoptotic activities of both Linsitinib‐resistant and ‐sensitive cell lines treated with Linsitinib and JSH‐23 alone or in combination. As expected, a single‐regimen therapy of JSH‐23 did not work well, but a combination therapy of both Linsitinib and JSH‐23 demonstrated a significant synergy in induction of apoptosis, as well as effective reduction in cell viability and colony formation in Linsitinib‐resistant cell lines. However, no difference was found in Linsitinib‐sensitive cells when they were treated with single or double regimens. Treatment with both Linsitinib and JSH‐23 exhibited increased efficacy of Linsitinib in Linsitinib‐resistant cells, indicating that targeting on both IGF‐1R and NF‐*κ*B may generate a promising therapeutic effect on ESCC.

To sum up, our study suggests that the intrinsic resistance of ESCC to Linsitinib may be mediated by NF‐*κ*B activation. A combined therapy that targets both IGF‐1R and NF‐*κ*B may provide a novel strategy to overcome the ESCC's resistance to Linsitinib.

## Conclusions

The intrinsic resistance of ESCC to Linsitinib may be mediated by NF‐*κ*B activation. A combined therapy that targets both IGF‐1R and NF‐*κ*B provides a novel strategy to overcome resistance to Linsitinib in ESCC.

## Conflict of Interest

The authors declare that they have no competing interests.
